# Interview with Prof. Dr. Jeffrey Drebin, President of the 2024 President Elect of the American Surgical Association

**DOI:** 10.1002/ags3.12882

**Published:** 2024-11-22

**Authors:** Koshi Mimori, Tsutomu Fujii, Masayuki Sho, Itaru Endo, Ken Shirabe, Yuko Kitagawa

**Affiliations:** ^1^ Department of Surgery Kyushu University Beppu Hospital Beppu Japan; ^2^ Department of Surgery and Science, Faculty of Medicine, Academic Assembly University of Toyama Toyama Japan; ^3^ Department of Surgery Nara Medical University Kashihara Japan; ^4^ Department of Gastroenterological Surgery Graduate School of Medicine, Yokohama City University Yokohama Japan; ^5^ Department of General Surgical Science Graduate School of Medicine, Gunma University Maebashi Japan; ^6^ Department of Surgery Keio University School of Medicine Shinjuku Japan

**Dr. Jeffrey A. Drebin** is Chair of the Department of Surgery at the Memorial Sloan Kettering Cancer Center and holds the Murray F. Brennan Endowed Chair. He is also Professor of Surgery at Weill Cornell Medical College. He was previously the John Rhea Barton Professor and Chair of the Department of Surgery at the Perelman School of Medicine of the University of Pennsylvania. He received his M.D. and Ph.D. degrees from Harvard, where his Ph.D. work involved the development of the first monoclonal antibodies targeting the HER2/neu oncogene. He subsequently performed his surgical training in General Surgery and a Fellowship in Surgical Oncology at the Johns Hopkins Hospital. Upon completing his clinical training, Dr. Drebin was recruited to Washington University School of Medicine in 1995, rising to Professor of Surgery and of Molecular Biology & Pharmacology in 2002.

In 2004 he was recruited to the University of Pennsylvania as Chief of the GI Surgery Division and in 2009 he became department Chair. At Washington University and at the University of Pennsylvania he established himself as a busy clinical surgeon, focusing on pancreaticobiliary, upper gastrointestinal, and liver surgery. He also established a successful translational research lab, receiving research support from the NIH, the Department of Defense, and the Burroughs Welcome fund for this work. Multiple surgery residents who worked in Dr. Drebin's laboratory have themselves gone on to successful academic surgical careers. He has published over 150 peer‐reviewed articles, chapters, editorials, and reviews, and has served on the editorial boards of multiple journals.

In his years at Memorial Sloan Kettering, Dr. Drebin has overseen a substantial increase in faculty size and corresponding increases in clinical activity and in NIH‐funded research. Dr. Drebin has been, and continues to be, involved with multiple organizations focusing on clinical surgery and cancer research. From 2016 to 2022 Dr. Drebin served as Recorder of the American Surgical Association. Dr. Drebin was named President‐Elect of the American Surgical Association in 2024. He is the Past President of the Society of Surgical Oncology, Past‐President of the Philadelphia Academy of Surgery, and Past‐President of the Society of Clinical Surgery. Dr. Drebin has previously served as a member of the Board of Scientific Advisors of the U.S. National Cancer Institute and on the Executive Committee of the American College of Surgeons Oncology Group. In 2013 he was elected to the National Academy of Medicine.

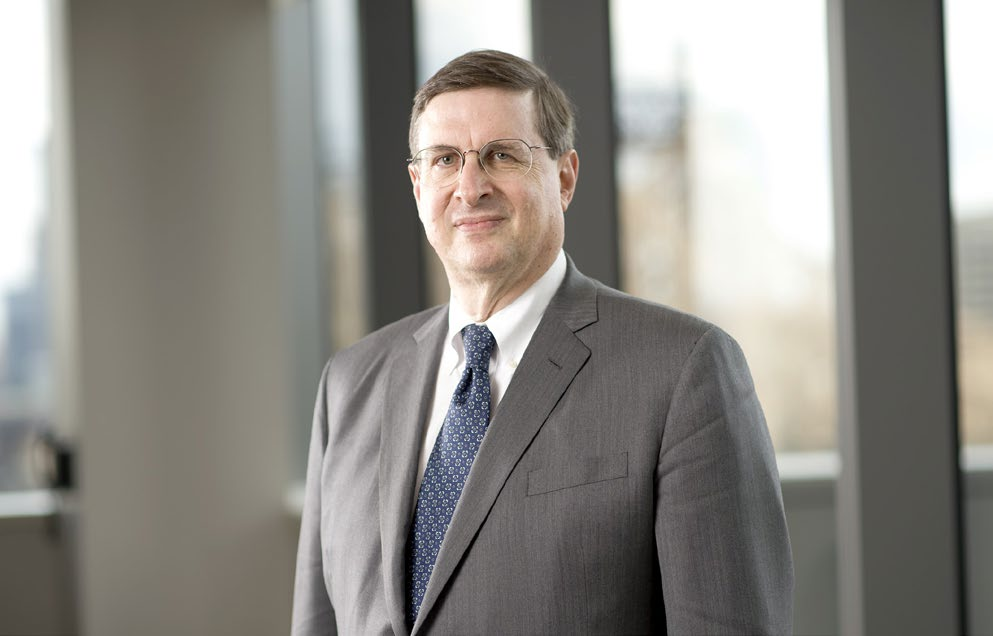

This is an interview with Prof. Jeffrey Drebin, a president elect of the American Surgical Association (ASA) and Chair at Memorial Sloan Kettering Cancer Center. Dr. Drebin recently published an article in ***Nature*** introducing an anti‐tumor effects of mRNA vaccine therapy for pancreatic cancer. In addition, he shared insights on the clinical topics, such as neoadjuvant chemotherapy for pancreatic cancer, approaches to treating elderly patients with pancreatic cancer and the radiotherapy for pancreatic cancer.

## 1 OPENING REMARK

Today, we are honored to have Professor Jeff Drebin, M.D., Ph.D., from Memorial Sloan Kettering Cancer Center, and the 2024 President Elect of the American Surgical Association, as our guest. We extend our sincere gratitude to Professor Drebin for taking time out of his busy schedule to join us at the 79th Annual Meeting of The Japanese Society of Gastroenterological Surgery. In addition, we deeply appreciate your participation and your contribution to the journal, *Annals of Gastroenterological Surgery*, an official journal of JSGS. Thank you very much, Professor Jeff.

### 1.1 Question 1: mRNA vaccine for pancreatic cancers

#### 1.1.1 Why are you interested in RNA vaccine for the treatment of pancreatic cancer cases?

*Recently, you published a paper in* Nature *about the mRNA vaccine for pancreatic cancer, and I wanted to ask you a few questions regarding this topic. It's a very hot topic (A brand new technology, mRNA vaccination for pancreatic cancer) and exciting paper, but I have several questions. The first one is about the personalized and rapid biosynthetic one, which boasts high efficiency with high immunogenicity neoantigens for the prevention of recurrence, particularly in the case of minimal residual disease (MRD) in pancreatic cancer. How do you determine the neoantigens*?

The mRNA vaccine work, I should give credit to my junior partner, Vinod Balachandran, who's a surgeon scientist at Memorial Sloan Kettering, who really has done a lot of the important laboratory work. Vinod not only has a very big and successful laboratory, he's also a pretty good surgeon. But Vinod's work had suggested that some patients who are long‐term survivors have evidence of a T‐cell response, and therefore raise the question of could we stimulate a T‐cell response with a vaccine, and in this case an mRNA vaccine.^1^

There may be other approaches that would work well. The first patient to get a vaccine in our trial was in December of 2019. Although we all know of mRNA vaccines from COVID and COVID vaccines, the first patient in New York to get it was in 2019. It happened to be my patient. But all of us within the group had patients in the trial. In December of 2019, he got his first dose of a vaccine made by BioNTech specific to his tumor.

The choice of mRNA had to do with the desire to be able to do this quickly, to have a broad antigenic representation from the tumor and some thoughts about safety. Although those were all things we evaluated in the phase one trial. Certainly, there are ongoing studies using a similar approach with peptide vaccines as well as DNA vaccines.

And so, I wouldn't claim this is the only approach that will work. But it was an approach BioNtech was interested in working on. Genentech participated in providing atezolizumab, the PD‐L1 inhibitor. And so, we sort of had both corporate support. We had our group, which was a relatively busy Pancreas Cancer Surgery Group.

And so, we did the trial.

#### 1.1.2 Are neoantigens derived from passenger mutations enough to eradicate pancreatic cancer?

*You identified individual personalized neoantigens from the mutations. However, there are four major genes that dominate pancreatic cancer. We have come to understand that these big four genes may overshadow the potential for other neoantigens to emerge. How do you decide on favorite neoantigens?*

In terms of antigenicity, pancreas cancer has a relatively low number of antigens.^2^ For most of our patients, they could fit up to 20 antigenic domains in the mRNA vaccine. Most of our patients didn't have 20 expressed neoantigens. In fact, I think the one who signed up for the trial couldn't participate because he had none. But most patients had anywhere from one to five or six. The responsiveness to the vaccine didn't correlate really with how many antigens they had interestingly.

The strategy that Genentech used was to look at both sequencing to identify mutations that could give rise to neoantigens, and then RNA sequencing to determine that they were expressed. The idea being that if the RNA was present, protein would be present, and therefore, there would be antigenic protein present in the cancer cell. Because the numbers were relatively small that fulfilled both criteria, it wasn't a question of having to narrow it down. We could put every mutation that was present in the patient onto their vaccine.

#### 1.1.3 Did you implement RNA vaccine treatment before the RNA vaccine for COVID‐19?

*I want to confirm that your work on the mRNA vaccine predates the COVID‐19 pandemic. While mRNA vaccines are now widely recognized due to their use in combating COVID‐19, was your system developed before the pandemic*?

Our mRNA vaccine was developed before the COVID‐19 pandemic. Everyone knows that mRNA vaccine is very famous for COVID‐19, but the system ignited before COVID‐19. Actually, the COVID vaccine became a perfect control.

Because after we started our trial and we saw some evidence that those who responded to the vaccine had a longer survival than those who didn't, one interpretation may be those who responded have a good immune system. Those who don't respond have a bad immune system. It's not the vaccine. The vaccine is just a selection for those who have a good immune system. But all of them got COVID vaccine.

The COVID vaccine response in the pancreas tumor non‐responders was as good as it was in the pancreas tumor responders. Their immune systems were equivalent. There was no difference in their ability to react to the COVID vaccine. It was simply that they didn't react to their own tumor vaccine in some patients.

#### 1.1.4 Are there reasons to use the vaccine therapy as an adjuvant treatment after surgery?

*Pancreatic cancer is a very challenging target because it is often surrounded by non‐inflamed tissue, CAFs, or fibroblasts. Your system seems highly effective since it targets the cancer after a pancreatectomy, which should enhance its effectiveness. Is my understanding correct*?

This is an adjuvant vaccine. We are talking about trying to put together a trial of a neoadjuvant vaccine. As you point out, one of the concerns in pancreas cancer is that the microenvironment of the tumor may suppress the immune infiltration of T cells or the function of T cells. And so, by doing this in an adjuvant setting, we have removed the tumor and it is really a minimal residual disease that we're hoping to treat. That may not have as many problems in terms of the microenvironment.

#### 1.1.5 What is the next step of the RNA vaccine

*Can your system be implemented on a global scale? Could you elaborate on how we can move forward with this*?

The next step—this was a phase one trial. It was really to say, could we do this? Our goal was to get the vaccine into patients within 10 weeks and we succeeded. The goal was to make sure that it didn't make people sick. We succeeded in this before the COVID vaccine, so we didn't know that an mRNA vaccine would be relatively safe. Then, the sign of efficacy was very encouraging. But again, responder versus non‐responder is a poor indication of whether a treatment ultimately will work.

And so, to validate this, we're now conducting a prospective randomized trial in patients who have pancreaticoduodenectomy and will either get standard adjuvant chemotherapy or chemotherapy plus the vaccine. Now, it's only in pancreaticoduodenectomy patients, because one thing that came out in the phase one study was pancreaticoduodenectomy patients were more likely to respond to the vaccine and patients who had a distal pancreatectomy with splenectomy were less likely to respond to it. We now know from animal studies that splenectomy may decrease responsiveness to the vaccine.^1^

#### 1.1.6 Is there a global tactic for this vaccine?

*We hope that pancreatic cancer patients worldwide will be able to access your system. Are there any plans to expand this treatment globally*?

The trial is a worldwide trial. I don't know that any sites are open in Japan yet, but if there are places that are interested, I suspect BioNTech would be happy to support them. I was in Korea 3 or 4 months ago and actually they have one site now that's open and it's open at a bunch of places in the United States. We hope to accrue several hundred patients for the phase two study if the results are good. One of the things that makes pancreas cancer research quicker is that if patients don't respond, they tend to not do well, and you get an answer quickly. If there is good evidence of efficacy at that point, I think it'll become a standard.

#### 1.1.7 What happened to the cases that showed a good response in the study?

*I'm very interested in the current status because, in the* Nature *paper, the patient who had a good response showed 100% survival. What is the situation now?*

The number we are now the 4 to 5‐year mark, with two relapses among the eight cases, both showing a good response. I believe they're both still alive, but I think two have had a relapse. Six out of eight are still relapse‐free at 3, 4, or 5 years. Whereas 100% of the non‐responders relapsed.

#### 1.1.8 Does the microbiome affect the efficacy of the RNA vaccine?

*Recently, some papers have shown that microbiota can influence immunotherapy. Do you have any data on whether the mRNA vaccination is affected by dysbiosis or microbiome*?

I don't have data, but I think it's an excellent question. I suspect as we know, the checkpoint inhibitor responses are affected by the microbiota. I suspect there will be something, but I don't have an answer.

### 1.2 Question 2: Neoadjuvant chemo for pancreatic cancer

#### 1.2.1 When is adjuvant chemotherapy indicated for pancreatic cancer?

*Next, I'd like to ask a more clinically relevant question. Do you believe that neoadjuvant chemotherapy is necessary for all resectable pancreatic cancers, not just borderline resectable cases*?

I don't (believe it). Within our group of seven HPB surgeons, there's a range of opinions. At Memorial, I think there are still some patients who go straight to surgery, some who get neoadjuvant. My personal practice is if there's borderline disease, if there's even venous involvement, that would require a major vein resection with interposition grafting, and there's a chance that it'll respond in a way that minimizes that or allows a side bite of the vein, rather than a total segmental, long segmental resection, we'll do neoadjuvant. But a patient with a disease well away from arteries and veins and no other problem, we'll take them straight to surgery.

#### 1.2.2 How would you improve outcomes for stage one pancreatic cancer patients?

*Well, I personally believe that neoadjuvant chemotherapy might be beneficial even for stage one pancreatic cancer because, compared to breast or colon cancer, stage one pancreatic cancer still has a less favorable outcome. After surgery, even adjuvant chemotherapy may not be sufficient for stage one pancreatic cancer. How would you improve outcomes for stage one pancreatic cancer patients*?

I don't disagree with you at all. I think pancreas cancer—there is no cancer, maybe other than melanoma, which a 1‐centimeter tumor is so bad. One‐centimeter lung cancer, 1‐centimeter colon cancer, gastric cancer, breast cancer, these are cured 80%, 90% of the time.

Pancreas cancer, less than 50%. Clearly, it's an advanced disease in many cases. For me, the distinction is that if you look carefully at the neoadjuvant data, there are patients who don't have tumor progression and yet never have surgery.

You have to look carefully. It's not something people advertise, because it's hard to explain.

But about 10% or 15% of people are made sick enough by the neoadjuvant therapy that they never come to surgery. And so, you have to trade off those patients with the fact that you avoid surgery in patients who are going to have early recurrence after surgery, which we've all seen and is very disappointing, of course.

I take your point that it's a systemic disease (cause this is what he said). I think it's critical that people get chemotherapy. I'm not so sure that whether you get it upfront or get it at the end will make a difference. As you know, there are studies going on to try to address that in a randomized fashion,^3^ but I don't think we have the answers. Although there have been studies, PREOPANC from the Netherlands^4^ was one that suggests that neoadjuvant is better.

There was also a recent study, the NorPACT Study,^5^, ^6^ which suggested neoadjuvant actually had a higher failure to get to surgery and did not improve overall survival on an intention to treat. I suspect if you ask five surgeons, you'll get five different answers.

Many institutions in the United States have taken the approach of everyone should get it, including some very prestigious and high‐volume centers. I don't disagree that would be one way to interpret the data. I understand in many centers in Japan, that's standard. I don't have a reason to say it shouldn't be. We tend to take a little bit more of an individualized view and say that if people are clearly resectable, with no evidence of vascular involvement, we go to surgery. Except for one of my partners who would do neoadjuvant. And in general, our patients get FOLFIRINOX for eight cycles.

When I do it, I tell people, we'll reassess at the end of 2 months, so four cycles. If they're having a response by CA19‐9, and by imaging, the tumor has responded dramatically, we may do surgery at 2 months.

In my experience, it's not that common to see dramatic tumor shrinkage at 2 months. Markers may go down better. It takes longer, I think, for the fibrosis to resolve. Even if the cancer cells are dead, the fibrous scar is still there. It's more common if I'm doing it for a reason, to get it away from an artery, to make a vein resection a little simpler, we would more commonly do 4 months. At 4 months, people are often getting a little worn out by FOLFIRINOX, and so we give them a break by doing a Whipple operation.

#### 1.2.3 What is the optimal indication of neoadjuvant therapy for resectable pancreatic cancer?

*In your or your colleagues' practice, what is the indication of neoadjuvant therapy for resectable pancreatic cancer*?

I don't do neoadjuvant for resectable. If a patient says to me, could I have it? My doctor says I should have it. I won't tell them that it's wrong. But in general, in my experience—and we did a neoadjuvant trial when I was at the University of Pennsylvania—I had patients who refused to participate in the trial because they said, I just want my tumor out. I don't want (take any extra steps or add any risks) —there's a—it's not logical. I don't think it's sensible, but I think there's a visceral (need to eliminate it immediately) —I have cancer. Get it out of me as soon as possible. In general, most patients don't argue if you say you can take it out. It's the opposite, really. When you say, I would like to do neoadjuvant and the patient says, can't you take it out first, doctor?

#### 1.2.4 What are the meanings of biological resectable and biological borderline?

*You mentioned anatomically resectable pancreatic cancer, but recently, some researchers have referred to biologically resectable or biologically borderline cancer*.

Certainly, if the CA 19‐9 is elevated, even if there appears to be—and somewhere between 500 and 1000 is my personal benchmark. If it's elevated at that level or higher, even if the tumor looks resectable, I would send them for neoadjuvant. That's a good point, thank you. I didn't point that out. I think if the markers are very high, as was noted earlier, pancreas cancer is almost always a systemic illness at diagnosis, and markers that high are going to predict early relapse. And so, we would do neoadjuvant in that setting.

#### 1.2.5 Does liquid biopsy for MRD apply to the practical clinical work?

*Since the Memorial is top of the cancer centers in the United States, do you use liquid biopsy or something for the indication of biological malignancy of the pancreatic cancer*?

We do have a liquid biopsy program. We were one of the first places to do routine sequencing of all of our tumors. We have over 100 000 sequences of all types at Memorial now. But the liquid biopsy, we tend not to use for decision making. It has been used for minimal residual disease status after surgery. In fact, there was a trial that was led by Eileen O'Reilly, who is our Chief of Medical Oncology, focusing on the pancreas. Eileen did a study looking at a vaccine approach for MRD, minimal residual disease defined either by persistent elevation of CA 19‐9 or persistent positive liquid biopsy, even with negative imaging.^7^ This was a KRAS vaccine trial that was also very encouraging.

#### 1.2.6 What is the regimen of adjuvant therapy in patients who underwent neoadjuvant FOLFIRINOX?

We have good data that adjuvant therapy requires 6 months.^8^ Less than 6 months has a higher relapse rate than patients who get a full 6 months, mostly from the British, from Neoptolemus and the European group. We tend to think of 6 months total as an important number. We don't have a lot of data for this; but if someone receives 2 months before, we tend to do 4 months after. If someone receives 4 months before, they will get 2 months after. If someone is doing well and wants to complete all 6 months before having surgery, we certainly do some total neoadjuvant. Most patients sort of get worn out before 6 months, but we think there's something important in 6 months, but I can't tell you. There have been really good studies in the neoadjuvant into adjuvant.

The other thing is to look at the pathology when you resect the patient. I think patients who have nodal disease, persistent after neoadjuvant, my bias is that's a bad finding. Patients who have very little evidence of tumor response histologically, when the tumor is resected, again, maybe even should have a different adjuvant therapy if the neoadjuvant didn't show much effect. I think you have to tailor this to the patients.

#### 1.2.7 How do you or the doctors of United States think about the nab‐paclitaxel^9^?

I should say at the outset that Dan Von Hoff, who led the global trial that led to, was my co‐leader of the Stand Up to Cancer group, and that was one of the projects that we supported.^10^ I've been a gem and nab‐paclitaxel supporter from early, and in fact, at the University of Pennsylvania, where I was until 8 years ago when I came to Memorial, I would say more patients got gem and nab‐paclitaxel than FOLFIRINOX.

For one thing, if you're going to do trials of chemotherapy plus some other agent, because gem and nab‐paclitaxel may be a little better tolerated, it's easier to combine with another form of treatment. That being said, our oncologists tend to go with FOLFIRINOX as their first line, whether in the neoadjuvant or the adjuvant setting. That's maybe an institutional preference as much as anything.

### 1.3 Question 3: Surgery in elderly patients

#### 1.3.1 How do you approach elderly patients with pancreatic cancers?

*As you know, recently, the number of elderly patients undergoing surgery has been increasing. Even octogenarians or patients over 90 are receiving pancreaticoduodenectomy. We believe that the postoperative complication rate and mortality are similar to those of younger patients. However, there must be careful patient selection. Some European papers have also mentioned the lower efficacy of adjuvant chemotherapy in elderly patient*.^11^ *I think this difference contributes to the disparity in survival between younger and older patient groups*.

I think this is an increasing problem. We know that pancreas cancer is a disease of aging. At least in the United States, it doubles every decade of life between the 40s and the 90s. We see it rarely in people in their 40s, but not uncommonly in the 80s and 90s.

You raised the question about chemotherapy, whether neoadjuvant or adjuvant. I think it's often the case that oncologists are concerned with giving either FOLFIRINOX or gem and nab‐paclitaxel to elderly patients. And so, it becomes a challenge. I think—I quote my patients as having increased mortality, but our overall mortality rate is pretty low. And so, the number is 1% to 2%. And so, I tell them it's 2% to 4%, so a bit higher, but in general, they're unlikely to get full‐dose chemotherapy.

Radiation therapy, this is really where radiation therapy may play a role in palliating people and buying a bit of time. But I think sometimes surgery is really the only alternative. And so, if you can do it safely, you have to be careful. You want to pick the patient who will tolerate a big operation. I think we would be much more hesitant to do a major vascular reconstruction as opposed to a relatively simple, straightforward, quick operation.

#### 1.3.2 Do you have some indication of a major operation?

Again, I think what I usually ask patients is what they're (capable of) —I don't routinely do stress testing or cardiac workup unless they have a history. I ask them about how mobile they are. I had a lady who was 89 years old. I said, can you walk a flight of stairs? She said, “I live in a four‐story walkup, but our laundry is in the basement, so I walk five floors when I carry the laundry basket up and down.” She went home in 8 days. I knew she was going to do well after pancreaticoduodenectomy.

I think you have to pick—if she had told me, I can't walk a flight of stairs without getting out of breath, and I have home oxygen, or I'm on four different heart medicines, then I think you need to be more careful and pick the patients carefully.

#### 1.3.3 How do you improve the postoperative period after pancreatic cancer surgery?

*Some Japanese researchers advocate the usefulness of both preoperative and postoperative rehabilitation—early rehabilitation, including ERAS (Enhanced Recovery After Surgery) and early recovery. However, in the United States, the postoperative hospital stay is often very short. Are there any measures to improve the postoperative period?*

Yes. I think prehab has not caught on very much. One of the challenges, if you're going to do neoadjuvant, I think that's a good opportunity to also do prehabilitation. If you're not going to do it, there's not too much evidence. It's like nutritional supplementation. You need weeks to really impact the patient, not a few days. And so, I'm not sure prehabilitation for a week and then doing their. If you're going to spend 2 months doing neoadjuvant, I think it's a good idea. But in general, if you're going to do surgery (of pancreaticoduodenectomy) first, probably you just need to do surgery.

Again, the length of stay, on average, is 7–10 days in our group. I wouldn't say it necessarily correlates that strongly with age. Again, I always say if I have an 80‐year‐old woman and a 50‐year‐old man, the 80‐year‐old woman will go home sooner every time. It's not just age— some of that's selection, because there are very few 50‐year‐olds I won't operate on. There are 80‐year‐olds who I would say no. Some of it's selection.

#### 1.3.4 What are your thoughts on neoadjuvant or adjuvant therapy in elderly patients with pancreatic cancer?

Yes. It's important. Since we know that surgery by itself does not do very well, that really multimodality therapy is critical for everyone to have the best long‐term survival. I think it's often the case that our oncologists don't want to treat with neoadjuvant, but if they come through a big operation, okay, it gives them a little more confidence to then give adjuvant. That's, again, our institution. I won't say that's a national standard. But certainly, there are places that do routine neoadjuvant, even in older patients.

#### 1.3.5 What is the optimal adjuvant chemotherapy for elderly patients?

*Actually, we previously published a paper comparing chemotherapy alone versus surgery in patients over the age of 80. In that study, we found that the completion of adjuvant chemotherapy is critical for long‐term survival. As you mentioned, adjuvant chemotherapy may be necessary for long‐term survival in very elderly patients. But in that case, what would you recommend for adjuvant chemotherapy—still FOLFIRINOX*?

One of the things—I don't get any support from any pharmaceutical company, so this is not (about promoting any specific treatment) —and I'm not a medical oncologist, so I don't have to give any chemotherapy. But I think the studies of gemcitabine‐capecitabine, gem‐cape, had results markedly better than single‐agent gem and not that much worse than FOLFIRINOX or gem and nab‐paclitaxel in the adjuvant setting. In older patients, that's actually where I try to steer the oncologist. What they'll often do is do a cycle of gem alone, and if the patient tolerates it, then add the capecitabine.

I had a 90‐year‐old man. Again, it shows the selection. He lived about six blocks from where the oncologist's office was, and he would walk over to get his chemotherapy and walk back to his apartment after his Whipple operation and got 6 months of adjuvant gem‐cape.

I think, again, if you select the patients, recognize that it's not only FOLFIRINOX or gem and nab‐paclitaxel, there is another factor. Even single‐agent gemcitabine has some benefits. I have a 93‐year‐old who I did a Whipple on in the last few months who's now getting single‐agent gem. Doing fine with it. The average survival of a 90‐year‐old is only 4 years, even if they die of pancreas cancer. Hopefully, we'll at least get some of that.

### 1.4 Question 4: Radiotherapy or ablative radiation therapy for pancreatic cancer

#### 1.4.1 How do you think about radiotherapy or the ablative therapy for local control?

I think, and I should say that I've been taught by our radiation oncologists, it's not something that I practice, but I have seen a lot of patients now. I think one of the things we know is that radiation therapy can cure cancer, but the doses required generally will kill the patient. And so, the trick is to find a dose of radiation that will inhibit the cancer but not hurt the patient.

Ablative techniques are really an approach to delivering a higher dose to the tumor without doing too much damage to surrounding tissues. In radiation therapy, there's been a great deal of focus on the type of particle, whether it's traditional gamma, whether it's proton therapy, carbon ion, various ways of focusing the beam, intensity‐modulated radiation therapy, stereotactic body radiation therapy. Probably, these are not as important as actually the dose.

Regardless of the particle type, it's critical to get a dose of approximately 100 grays or 10 000 centigrays, whereas the prior dose that we traditionally gave for pancreas was around 5000, 5400. That dose is really a palliative dose, and there's pretty good evidence that will slow tumors down, but rarely result in good long‐term control.

The other thing is that we know that pancreas cancers in the majority of patients have perineural invasion, they have lymphatic invasion, and so the tumor is not a very small, focused area. It's somewhat diffuse. Stereotactic approaches have the advantage of being tremendously focused. But for a disease that's going to be on the edges, that may not be a good thing. In fact, there's data that Stereotactic Body Radiation Therapy may be inferior to more traditional approaches because of the inability to cover the border.^12^

And so, the ablative approaches that our radiation therapists use is designed to get up to about 100 gray (Gy) of the tumor and to cover the surrounding area, and yet to do it with a dose that doesn't cause patients to get very sick, and that we are doing more and more for local control, either preoperatively or in patients who can't have surgery.

#### 1.4.2 How do you apply the chemoradiotherapy for pancreatic cancer?

*In Japan, chemoradiotherapy is usually indicated only for locally advanced pancreatic cancer. What are your thoughts on its indication for resectable pancreatic cancer?*

We actually have a similar belief. I wouldn't say it's universal. There are several very good high‐volume centers led by very experienced surgeons in which radiation therapy, particularly in the neoadjuvant setting, is routine. MD Anderson, of course, pioneered that for many, many years, and a number of their disciples that are at other institutions where that would be standard. That's not our approach. For patients, even if they have a neoadjuvant approach, neoadjuvant chemotherapy, we don't do radiation therapy unless we're in a situation of locally advanced disease or borderline disease that's not responding to chemotherapy.

But the ablative radiation therapy does not prevent subsequent surgery. On occasion, that becomes a step. Patients get routinely 3–4 months of chemotherapy. If they haven't responded well enough, they may get ablative radiation therapy and then still potentially have surgery or not.

Now, for patients with locally advanced disease, and I think all surgeons know, there are patients who know that response to chemotherapy is going to result in a tumor that's completely encasing the celiac or the SMA going away. In those patients, ablative radiation can result in fairly impressive long‐term local control. Of course, it does nothing for systemic disease. But increasingly, I just saw a patient with this problem this week.

Increasingly, we're being asked to do palliative hepaticojejunostomies 2 or 3 years after a patient with locally advanced disease had a wall stent placed, got chemo, got ablative radiation, and now their wall stent has clogged up, they've had it cleaned out and had a new stent put inside it, and now that's gotten clogged up and they keep coming back with cholangitis, and we're asked to do a hepaticojejunostomy 2, 3 years after their diagnosis, knowing that cancer is still there, but it's not growing or progressing. I've done a few of them and several of my partners have done some.

Again, operating on patients with locally advanced disease is not something I'm encouraging. But I think it's an indication of how, in some patients, ablative radiation really buys people time. We published a paper in which we looked at about 100 patients who had ablative radiation for locally advanced or borderline disease versus 100 who had surgery.

The groups weren't really comparable because, of course, the surgical group tended to be borderline, the radiation group tended to be locally advanced, arterial involvement in the vast majority in the radiation group, in the vast minority in the surgical group. But we then looked at the pattern of recurrence, and actually, the local control rate with radiation was better than with surgery.

Now, metastasis was more common if you left the tumor there and didn't take it out, so 2, 3, 4 years later, you leave live cancer, it's not surprising. Furthermore, we know that patients who have locally advanced disease are more likely to have metastatic disease than borderline patients. These groups weren't totally comparable. But it's interesting that the local control rate is remarkably good.

The other thing we sometimes do is we'll have a patient who, a year or two after a pancreaticoduodenectomy, has local recurrence. Frequently, it's at the artery. The margin that's most commonly found is the uncinate margin, and they've now got local recurrence. CA 19‐9 may be going up. No metastasis. Of course, they always ask, can you operate? The answer is, no, we can't. But they can often do ablative treatment for that. That appears to, again, at least hold it in check for years. That may be a good, not a curative approach, but a palliative approach.

#### 1.4.3 What do you think about the optimal regimen of chemotherapy in combination with radiation?

Our standard is 4 months of chemotherapy. In part, sort of, I think it gets back to the question of neoadjuvant. One of the advantages of neoadjuvant chemotherapy is there's a group of patients who are going to have early systemic relapse after surgery, if you do a surgery‐first approach. I think the same is true of radiation. You'd hate to give someone a course of radiation therapy.

During that, they sometimes use capecitabine as a potentiator, but they don't get full‐dose chemotherapy during the radiation. If you assume that they have locally advanced disease, they certainly have microscopic systemic disease. They should get chemotherapy first, and then if they are able to get radiation after that, that becomes a way to get the majority of patients out around 2–3 years, which is not that different than how they do with surgery.

#### 1.4.4 Do you think that using an immune checkpoint inhibitor (ICI) together with ablation is appropriate?

I think one of the questions that we really haven't answered, in animals, there's very good data of a so‐called abscopal effect. You radiate the tumor. It releases antigens. The checkpoint inhibitor then can stimulate and help the immune system. I don't think we have great evidence of that in pancreas cancer.

In humans. But I don't think it's a, I think it's well worth studying, and I know there are trials going on.

## 2 CLOSING REMARK

We're going to wrap up this conversation. Thank you very much for your insightful elaboration and explanation about pancreatic cancer. We face significant challenges in curing pancreatic cancer, but today's discussion with Professor Drebin provides a glimmer of hope. In the future, we may be able to eradicate pancreatic cancer. Thank you very much for your active discussion.

### AUTHOR CONTRIBUTIONS

Koshi Mimori: writing manuscript; Tsutomu Fujii: Interviewer; Masayuki Sho: Interviewer; Itaru Endo: Interviewer; Ken Shirabe: Supervise study; Yuko Kitagawa: Principle investigator.
